# The Decision-Making Process for Palliative Sedation for Patients with Advanced Cancer–Analysis from a Systematic Review of Prospective Studies

**DOI:** 10.3390/cancers14020301

**Published:** 2022-01-08

**Authors:** Alazne Belar, Maria Arantzamendi, Johan Menten, Sheila Payne, Jeroen Hasselaar, Carlos Centeno

**Affiliations:** 1 Institute for Culture and Society-Atlantes, Universidad de Navarra, 31009 Pamplona, Spain; abelar@unav.es (A.B.); ccenteno@unav.es (C.C.); 2 IdiSNA—Instituto de Investigacion Sanitaria de Navarra, 31008 Pamplona, Spain; 3 Department of Oncology, Laboratory of Experimental Radiotherapy, Katholieke Universiteit, 3000 Leuven, Belgium; johan.menten@uzleuven.be; 4 Division of Health Research, Lancaster University, Lancaster LA1 4YW, UK; s.a.payne@lancaster.ac.uk; 5 Department of Anaesthesiology, Pain and Palliative Medicine, Radboud University Medical Centre, 6525 GA Nijmegen, The Netherlands; Jeroen.Hasselaar@radboudumc.nl; 6 Departamento Medicina Paliativa, Clínica Universidad de Navarra, 31001 Pamplona, Spain

**Keywords:** palliative sedation, sedation, palliative medicine, palliative care, terminal care, terminally ill, hospice care, decision making, prospective studies, secondary analysis

## Abstract

**Simple Summary:**

The involvement of patients in decision making about their healthcare plans is being emphasized, but little is known how the decision making on palliative sedation is conducted and who are involved in. The aim of this study is to understand how decisions about palliative sedation are taken. The results may help to understand the reality of this decision-making considering when and by whom the decision-making process is initiated, patient involvement, family involvement and healthcare involvement. This may contribute to identifying aspects that should be improved.

**Abstract:**

Background. The involvement of patients in decision making about their healthcare plans is being emphasized. In the context of palliative sedation, it is unclear how these decisions are made and who are involved in. The aim of the study is to understand how this decision-making is taken. Method. Information from a systematic review on clinical aspects of palliative sedation prospective studies were included. PubMed, CINAHL, Cochrane, MEDLINE, and EMBASE were searched (January 2014–December 2019). Data extraction and analysis regarded: (a) When and by whom the decision-making process is initiated; (b) patient involvement; (c) family involvement and (d) healthcare involvement. Results. Data about decision making were reported in 8/10 included articles. Palliative sedation was reported in 1137 patients (only 16 of them were non-cancer). Palliative sedation was introduced by the palliative care team during the disease process, at admission, or when patients experienced refractory symptoms. Only two studies explicitly mentioned the involvement of patients in decision making. Co-decision between families and the regular health care professionals was usual, and the health care professionals involved had been working in palliative care services. Conclusion. Patient participation in decision making appeared to be compromised by limited physical or cognitive capacity and family participation is described. The possibility of palliative sedation should be discussed earlier in the disease process.

## 1. Introduction

Since 2010 the importance of having end-of-life conversations with patients has increasingly been recognized, fostering shared decision making, especially for persons with advanced progressive life-threatening disease [1,2]. A systematic review has shown that patients preferred sharing decision making in 71% of the studies from the year 2000 to present, compared to 50% of studies before 2000 [3]. Shared decision making engages patients in their illness trajectory, encouraging their participation on deciding about treatment, or follow-up. It integrates the best available evidence and patients’ values and preferences during the illness [2]. However, studies identified a subset of patients who wanted to delegate decisions. These patients may not want to take the responsibility of being involved in medical decision making for different reasons [3]. Therefore, it is important for healthcare professionals to start regular communication about decision making preferences during the disease trajectory.

This can be undertaken as advanced care planning [4] or serious illness conversations [5], but the objective is the same: to explore the personal preferences in dialogue regarding the present and future care plan [4]. In these conversations, different aspects of care are discussed and may include conversations about symptom control, cardiopulmonary resuscitation, ventilator support, the use or withholding of antibiotics and artificial nutrition and hydration, or palliative sedation for the relief of refractory suffering [6].

According to the European Association for Palliative Care (EAPC), palliative sedation refers to the “controlled use of medications intended to induce a decreased or absent state of awareness (unconsciousness) in order to alleviate the burden of suffering that would otherwise be intractable, in a manner that is ethically acceptable to patient, family and healthcare providers” [6] (p. 581).

In some cases, the patient’s ability to participate in decision making at that moment may be affected, such as in the presence of non-responsive agitated delirium or in emergency situations. The administration of palliative sedation medication should be proportional to that required to relieve the patient’s suffering as much as needed for the patient and can be administered intermittently or continuously. Palliative sedation can be light, intermediate or deep depending on the levels necessary to reach the patient’s desired comfort [7]. All of this can lead to situations in which the patient after the administration of sedatives has no longer the possibility to interact adequately with the environment [8,9,10,11], or share their preference for sedation.

In cases where the patient had not in advance discussed his/her preferences about palliative sedation with the family and the professionals, its use may lead to distress both in the family [12,13] and in the professional team [14,15]. Due to that, in certain situations, doubts may arise within the team about the suitability of palliative sedation and the care preferences of patients.

Prospective studies on palliative sedation have been concerned with analyzing various (clinical) aspects of medical practice. As far as we know, none have addressed, as a primary objective, the decision-making process performed before administering palliative sedation. To obtain a preliminary understanding of how the sedation process occurs in clinical practice in different settings and countries, this study aims to analyze the information available in prospective studies on decision-making process in palliative sedation.

## 2. Materials and Methods

This study analyzes the data available on the decision-making process of palliative sedation reported in recent prospective studies included in a systematic review on clinical aspects of palliative sedation. 

The method of the review is already reported in a previous publication [16] and registered at PROSPERO (CRD42019136326) [16]. In summary, PubMed, CINAHL, Cochrane, MEDLINE, and EMBASE databases were searched from 2014 to 2019, combining the terms sedation, palliative care, and prospective (studies) (Table 1). 

During the analysis and writing of the systematic review, the researchers noted the limited and varied references to decision-making processes, in a topic that is very relevant due to the decrease or absence of consciousness, and it is consequences for relational capacity. It was therefore decided to conduct a secondary analysis with a focus on these aspects.

The secondary analysis enabled the articles and data selected for another purpose to be used to address a different research question [17,18]; in this case on decision making. The approach that was taken here was to reanalyze all of the dataset going back to the original articles while attending more specifically to ‘decision making’. The elements about the decision making about palliative sedation that the EAPC framework mentioned were considered in this re-analysis and data extraction process [6]: (1) When and by whom the decision-making process about palliative sedation is initiated; (2) patient involvement and; (3) family involvement in the decision-making process and (4) healthcare providers involvement in the decision-making process. A data template was built with these points and data were extracted from the included papers.

Data extraction was conducted by two researchers (AB and MA) with each researcher responsible for the data extraction of 50% of the articles and independently extracting data on 10% of the articles of the other reviewer, to ensure data extraction was undertaken rigorously [19]. A third researcher (CC) checked that all information concerning decision making was included in the template.

## 3. Results

**Figure 1:**
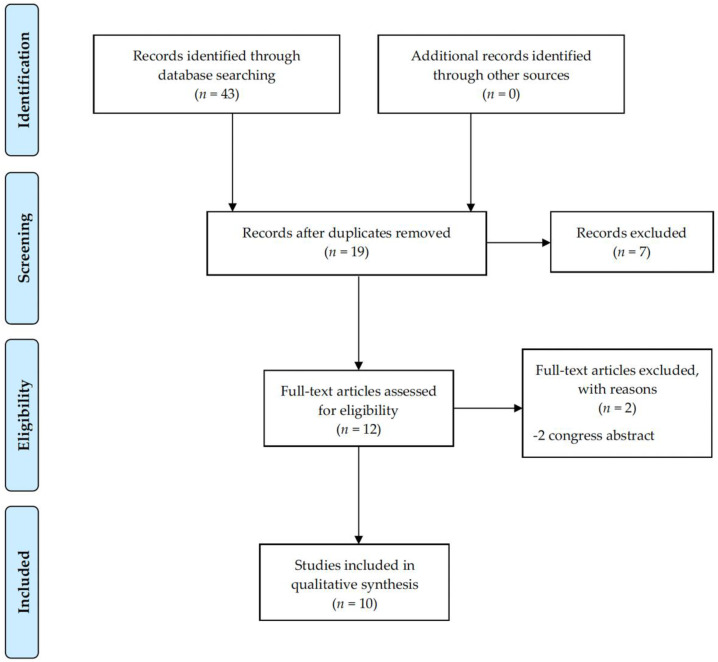
PRISMA flow chart from the search strategy.

In total, 43 articles were identified. Citation tracking and reference list checking did not add any additional records. After removing duplicates, and title and abstract screening, 12 full-text articles were assessed, resulting in ten articles eligible for the systematic review (Figure 1). 

Data about decision making was reported in eight out of ten articles [8,20,21,22,23,24,25,26], while the two other studies did not mention how decision making concerning palliative sedation was conducted [27,28]. Studies were conducted in Colombia [20], Italy [23,26], Japan [24,25], México [21] and The Netherlands. This last country published two articles from a single study [8,22]. All the studies used a prospective design, some were identified as observational [8,21,22,23], others as descriptive [20] or as longitudinal [23,27,28]. The type of palliative services included were: palliative care teams in hospitals [20,25], palliative home care service [23,24,25,26], in-patient hospices [8,22,23], nursing home-based palliative care units [8,22] and palliative care units in hospitals [21,24,25]. No differences were found regarding the decision-making process considering the settings that palliative sedation was conducted. The studies are based on samples of patients ranging from 24 [26] to 531 [23]. In total, sedation of 1137 patients were reported, and only 16 were patients with non-malignant diseases.

The results are reported considering the EAPC framework which mentions different aspects of the decision-making process. 

### 3.1. When and by Whom the Decision-Making Process about Palliative Sedation Is Initiated

Considering when palliative sedation was first discussed with patients, studies explicitly reported that decisions about administering palliative sedation arose when the patient is experiencing one or more refractory symptoms [20,21] and when it caused significant suffering [20]. In three out of ten studies the option of palliative sedation was sometimes previously discussed in the course of the disease trajectory [20] or at admission to a hospice or nursing home-based palliative care unit [8,22]. The decision-making process was led by the palliative care team [8,21,22,23,24,25] and in some cases specifically it was only led by the attending palliative medicine physician [8,21,22].

### 3.2. Patients’ Involvement

One study reports the impossibility of some patients being involved in decision making before administering palliative sedation due to their deteriorating clinical condition [26]. 

The other nine studies show limited patients’ involvement in the decision-making process. The two papers of Van Deijck et al., describing the same patient cohort, state that the discussion on palliative sedation to be included in the study could be carried out with the patient or their representative, but they do not indicate how many patients participated in this discussion [8,22].

The involvement of patients in decision making is also doubtful in another study [20] where they mention that specialists discussed with the patient and/or family -prior to sedation- the type of sedation that would be administered, and that sedation began after obtaining this consent [20]. In this study, 66 patients were sedated, but, although it is deduced that it was discussed with more than one patient, they do not provide any data explaining how many patients gave the consent [20]. 

Only one study mentions that palliative sedation was first proposed by 5% (*n* = 27) of the finally sedated patients although around one third (*n* = 177) of those who were sedated have expressed an opinion on palliative sedation [23]. Another study suggests patients’ involvement in the decision making as it mentions that the patient’s wish of palliative sedation needed to be explicitly or sufficiently presumed, together with the family desire to achieve greater symptom control before palliative sedation was administered [24].

In this regard, it is noteworthy to point out that palliative sedation was administered proportionally until death [16]. The duration varied depending on the articles, but the median duration was 25 h and mean durations 40–70 h, which indicated the very last days of the patients [8,21,22,27]. 

### 3.3. Family Involvement

Two studies mention a co-decision process between families and professionals about administering palliative sedation because of the presence of refractory symptoms. This occurred in every sedated patient included in one study [21] or just in some sedated patients in the second study (30%, *n* = 7) [26]. The other 8 studies explained that only family members participated in the decision-making process due to the patients´ cognitive incapacity to participate [21,26]. This happened in 96% of cases where palliative sedation was administered [23]. Palliative sedation starts “once the patient (when possible) or family gave consent” [20].

Four of ten studies explain that the discussion about palliative sedation between family and healthcare professionals happened after consent in the healthcare team so that the decision-making process in the team was finalized when at some point family consent was given [20,21,23,25]. 

### 3.4. Healthcare Involvement

Palliative care team professionals were involved in the decision-making process [8,21,22,23,24,25]. In addition, all ten studies show that the monitoring of palliative sedation was undertaken by the palliative care professionals since all these studies were performed in palliative care services: palliative care unit [8,21,22,24], palliative care institutions [25], hospices [8,22,23] and palliative home care programs [23,26].

## 4. Discussion

Palliative sedation entails lowering consciousness by titrated medications to relieve suffering but at the same time reduces the possibility for meaningful interactions [8,9,10,11]. This may sometimes be distressing for patients and/or relatives and a particularly careful decision-making process is crucial [12,13]. 

The European Association for Palliative Care framework presents recommendations for the decision-making process indicating the importance of the active discussion about palliative sedation between patient, family and healthcare professional (6).

There is limited data on decision making provided in the selected prospective studies. It could be argued that this is because the objectives of the studies were unrelated to decision making. It could also be that the literature focus on decision making regarding the suitability of the treatment and type of sedation, thus, there is little evidence on the process and different participants involvement. This study may have not regarded yet in the line with the current discussion trend which encourage patients´ involvement [29,30]. This paper adds some evidence to this regard.

The analysis of the studies suggests that final consent for palliative sedation was requested just before sedation [21,23,24,25,26] and an early discussion on palliative sedation with patients´ took place during the earlier disease trajectory in only one study [20] or at the admission to the palliative care service in another study [8,21]. It suggests an attempt was made to consider patients perspectives but it is unclear how it was undertaken. Further efforts should be made to use a model of decision making between health care professional and patient as it is considered a quality indicator of palliative care [31] and shows consideration and also respect for patients’ autonomy [32]. Patients seem to be, in general, willing to participate in everyday care decisions, treatment-related medical decisions and end-of- life decisions [33]. Prerequisites for patient participation in shared decision making are interdisciplinary teamwork, open communication, good patient–healthcare professional relationship, a favorable environment and mutual information [33]. In the case of palliative sedation, our analysis shows that in 2 Italian papers it was impossible or even inappropriate to obtain consent directly from the patient [23,26]. This highlights the importance of timeliness of referrals to palliative care services and the origins of these as they may affect the discursive practices initiating decision making about palliative care options [34], among which is palliative sedation. A qualitative study on cancer patient’s involvement on decision making preceding continuous sedation in Belgium, the United Kingdom and the Netherlands describe a four-stage decision-making process: initiation, information exchange, deliberation and the decision to start the sedation [35]. These stages require time to be carried out and implies having earlier conversations about diagnosis and prognosis, which may be impossible if patients are referred at a very late stage to palliative care services. An observational longitudinal study about decision making conversations in palliative care highlighted the importance of consistently introducing decision making conversations early on and discussing future options of care [34]. They also emphasize the need to teach physicians about this, and the consequences of failing to do so [34].

In addition, our review highlighted that the conversation with patient or family tends to happen as a second step, as the first step was talking between professionals. This may seem controversial but there are different perspectives regarding decision making. These can range from the predominance of autonomy to paternalism with “intermediate” positions such as shared decision making [11]. It could also be needed as part of the clinical discussion about the refractoriness of the symptoms and the potential reasons for considering sedation. The physician’s knowledge and experience and practical skills in palliative sedation and the ethical considerations that may arise could also explain this [30,36]. This discussion among professionals could be regarded as positive because it involves an interdisciplinary and interprofessional and collective approach when reaching consensus, after all professionals have explained their own perspectives and arguments [33], which make the therapeutic option of palliative sedation more consistent and justified. 

However, in the assessment of refractory symptoms, team involvement is important for taking into account different professionals’ perspectives which can be enriching and contribute to the holistic care of the patient [6]. However, as it is shown in this study, it is not clear that these team discussions always happen within a holistic assessment approach. 

Differences in cultural perspectives, given that the studies emerge from several continents, require attention to terms such as paternalism and patient autonomy as they can be interpreted differently [37,38]. The findings of this study do not allow us to draw cultural differences due to the small sample of countries included. Integrating the patient´s personal and cultural perspectives into their therapeutic plans and decision-making process is key for personalized care [39]. This is a challenge as difficulties in talking about palliative sedation at admission are reported, because the health care team were unfamiliar with the values of the patient and family [40]. Decision making is not only about doing but also about how it is undertaken. A study suggests that most family members, unfamiliar with end-of-life discussions, were satisfied with the sedation and staff support but report that the explanation about the treatment was given on the same day that it started and missed so the many opportunities to discuss the treatment earlier in the disease trajectory [41]. The way the information and the process is managed needs to consider the patient’s clinical, cultural and social context [42]. 

The data suggest that advance care conversations about palliative sedation happen too late in the disease trajectory, that the involvement of patients seems to be minimal or absent due to their clinical or cognitive deterioration. It is a difficulty for clinicians as patients cannot communicate adequately to make decisions [43]. This makes it difficult or impossible to know for sure if the wishes of the patient are being respected, especially if there have been no early conservations about it. In this regard, it would be interesting to consider in future studies if the timing and the way in which the conversation about sedation is undertaken/performed, and its influence on future administration of palliative sedation. However, scientific literature describes some barriers for having advance care conversations, such as patients´ fear of confronting their death, professionals related difficulties to communicate about these issues [44,45] or identifying the opportune moment for starting shared decision making [46]. Otherwise, introducing earlier conversation about palliative sedation as a treatment resource during advance care conversations may reduce families [11,12] and health professionals’ distress [13,14] during and after palliative sedation. 

This study has some limitations including the absence of studies that focused on the process of decision making to start palliative sedation and the poor reporting of this in all the studies reviewed. The analysis was part of a review with a broader objective, so it could be that by not carrying out a specific search for this research question, not all the relevant literature about decision making on palliative sedation has been included but only that is obtained from recent prospective studies. The aims of the small number of studies included were not focused on decision making so it is possible that just a little data about decision making was presented in the articles. 

Data suggest the need to design alternatives to increase involvement of patients in the critical decisions needed at the end of life.

## 5. Conclusions

The decision-making process seems to be performed very or too late in the disease trajectory, which makes patient involvement limited or absent due to their clinical deterioration. Palliative sedation—where appropriate—should ideally be discussed with patients and family earlier in the disease process in the context of shared decision making. Team discussion and reaching consent is essential in the decision-making process and trained palliative care professionals should participate. 

## Figures and Tables

**Table 1:** Search strategy.

Database	Concepts and Combinations				
	Sedation		Palliative Care		Prospective
**PubMed**	Sedation (Title)	AND	Palliative care (MeSH)	AND	Prospective (MeSH)
**Medline (WoS)**	Sedation (Title)	AND	Palliative care (MeSH)	AND	“Prospective studies” (topic)
**Embase**	“Palliative sedation” (Title)	AND	“Palliative care” (Abstract)	AND	Prospective (All files)
**Cinahl**	Sedation (Title)	AND	“Palliative care” (Abstract)	AND	Prospective (abstract)
**Cochrane Library**	Sedation (Title, abstract, key word)	AND	“Palliative care” (Title, abstract, key word)	AND	Prospective

**MeSH:** Medical Subject Headings; WoS: Web of Science; **Limits:** English language; published between January 2014–September 2019.
